# Proceedings of the Sixty-Eighth Annual Convention of the Connecticut Med. Society

**Published:** 1860-07

**Authors:** 


Proceedings of the Sixty-eighth Annual Convention of the Connecticut Medical Society, held at Hartford, May 23d and 24th, 1860.

This is a volume of 72 pages, containing the list of officers; the address of the President, Dr. Ashbel Woodward, on Medical Ethics; an essay on Hygiene, by Dr. E. B. Haile, of Norwich; a Sanitary Report, by Dr. L. S. Wilcox, of Hartford; and Biographical Sketches of Drs. Benjamin Rogers, Joseph F. Jewett, Horatio Dow, James Morgan, Ambrose Ives, and Sturges Bulkley.

